# Navigating the Evolving Landscape of Cardiovascular Training and Education

**DOI:** 10.14797/mdcvj.1127

**Published:** 2022-06-03

**Authors:** Nadeen N. Faza, Stephen H. Little

**Affiliations:** 1 Department of Cardiology, Houston Methodist Hospital, Houston, Texas, US

**Keywords:** cardiovascular training, cardiovascular education, fellowship

Recent years have marked significant changes in cardiovascular training and the educational landscape. Pioneering research and innovations have transformed cardiovascular care and redefined training methods and goals, leading to the evolution of novel subspecialties. In addition, the widespread use of digital platforms has had an impact on medical literature dissemination and provided a unique venue for asynchronous teaching and learning. Focus has additionally shifted to ensure a more diverse workforce, which has proven to improve patient outcomes. Lastly, the COVID-19 pandemic has temporarily disrupted traditional training methods, thus allowing educators and trainees to develop and embrace novel educational strategies. We have dedicated this issue of the *Methodist DeBakey Cardiovascular Journal* to highlighting the various evolving aspects of cardiovascular training and education.

Cardiology is a rapidly growing field fueled by extensive research and innovations. This places extra emphasis on optimizing the educational processes during fellowship training. Drs. Natalie Stokes and Kathryn Berlacher launch this issue with their overview of the science of learning and the art of education. To help both fellows and educators, they provide examples and creative methods to demonstrate how the principles of adult learning can be used effectively for cardiology training.

While cardiovascular disease is the leading cause of maternal mortality in the United States, dedicated cardio-obstetrics training programs have not been well established to close this gap in care. Drs. Anum Minhas, Erin Michos, and coauthors shed light on cardio-obstetrics as a budding subspecialty, especially at a time when maternal mortality is rising and dedicated training pathways are lacking. In their review, they propose a specialized cardio-obstetrics training pathway that prepares trainees to provide comprehensive care to women before, during, and after pregnancy.

The cardiac critical care (CCU) landscape has drastically changed since the 1960s as advanced therapies are increasingly being offered to patients with cardiac disease. These include mechanical support devices, transcatheter structural heart disease procedures, high-risk percutaneous coronary interventions, and ablations. Therefore, the profile and complexity of patients being admitted to the CCU has changed, necessitating that critical care cardiologists are equipped with advanced skills to manage patients in this setting. Drs. Ann Gage, Andrew Higgins, and Ran Lee discuss the advent of cardiac critical care as a novel subspecialty of cardiovascular medicine.

The field of cardiovascular surgery also has witnessed remarkable changes over the past few years. With advances in surgical techniques and device technologies, outcomes after cardiovascular surgery have improved. With the advent of transcatheter structural heart interventions that have shifted the paradigm of managing patients with structural heart disease, cardiovascular surgeons are key members of the heart team. Drs. Quasim Al Abri and Moritz C. Wyler von Ballmoos review the current status and future of cardiovascular procedural training and evaluation. They also underscore the triad of required expertise to master the spectrum of therapeutic options in cardiovascular surgery: surgical expertise, catheter expertise, and imaging expertise.

Advances in multimodality imaging have not only revolutionized cardiovascular care but also have had a major impact on vascular surgery practices. Drs. Kavya Sinha, Trisha Roy, and colleagues describe the transition from “vascular surgeons” to “vascular specialists,” highlight the key role of imaging in vascular surgery, and identify vascular imaging-related gaps in knowledge. With the lack of formal training that includes modalities other than vascular ultrasound, the authors propose a dedicated advanced multimodality vascular imaging fellowship program to expand a vascular specialist’s toolbox that could result in offering personalized treatment plans.

Launching an academic career in cardiology can be daunting yet very rewarding. Drs. Nino Isakadze, Erin Michos, and coauthors delve into the challenges of starting a research career in cardiology and focus on the significance of effective mentorship and sponsorship. In their review, they also provide valuable advice to fellows-in-training and early-career cardiologists for finding their passion, niche, and research focus while embarking on an academic career in cardiology.

Given the rapid expansion and wide digital dissemination of medical literature, digital platforms have recently provided adjuncts to traditional educational resources. With the popularity of these platforms increasing among cardiologists and fellows-in-training, the “cardionerds” review the benefits and pitfalls of digital educational platforms. Drs. Gurleen Kaur, Daniel Ambinder, and Amit Goyal propose a three-pillared approach—consume, contribute, and create—to help fellows-in-training succeed as learners and educators in the digital era.

Another important aspect of cardiovascular disease fellowship training is diversity in the workforce, which results in improved clinical care, greater access to care in underserved communities, and enhanced cultural competence. Drs. Ingabire Grace Balinda and Nosheen Reza highlight the virtues of diversity set against the challenges encountered by women and underrepresented minority cardiologists. They also emphasize strategies to improve diversity in cardiology training programs.

The COVID-19 pandemic has undoubtedly changed the face of cardiovascular education and training. The disruptive obstacles posed by the pandemic have dictated creative solutions to ensure effective, uninterrupted cardiovascular training. In a review led by Dr. Hyeon-Ju Ali, we conclude this issue by reviewing the training challenges associated with the pandemic, how they were overcome, and lessons learned that have improved the quality of education and ensured trainee well-being.

We are grateful to our experts, who have provided up-to-date reviews on key topics that are pertinent to fellows-in-training and educators. We hope our readers enjoy the many highlights they have shared about key aspects of the cardiovascular training and educational landscape in 2022—and in the years ahead.

## Editor Biographies

The editorial team of the *Methodist DeBakey Cardiovascular Journal* express our sincerest thanks to Drs. Nadeen Faza and Stephen Little for their enthusiasm and expertise in directing this issue on cardiovascular training and education.

## Nadeen N. Faza, MD, FACC, FASE, FSCAI

**Figure:**
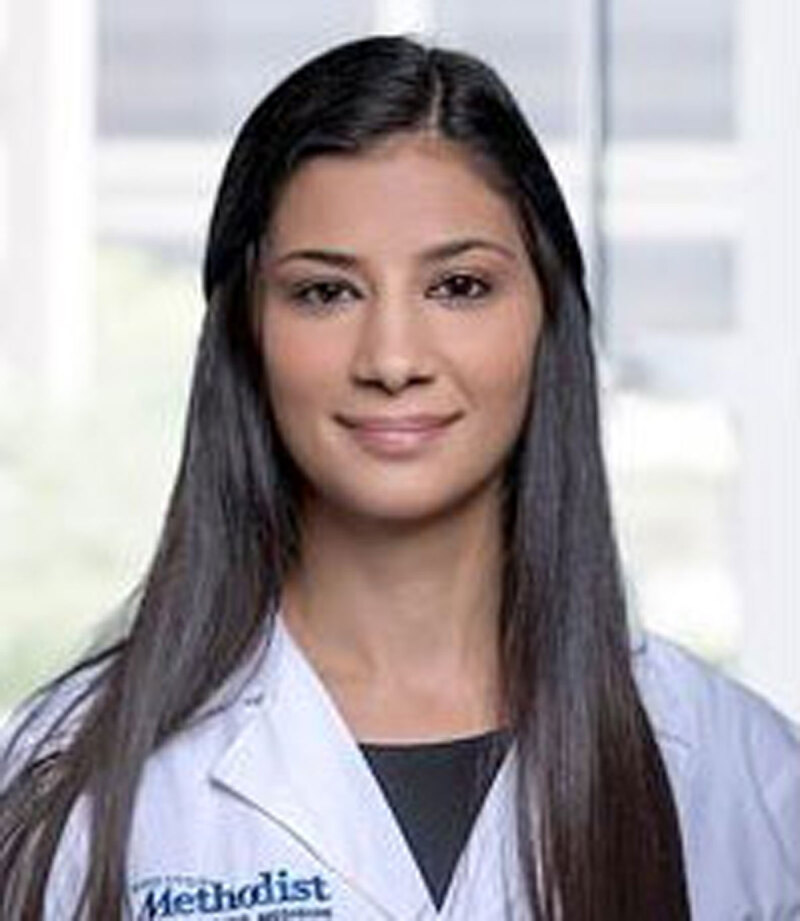


Dr. Faza is the associate program director for Houston Methodist’s Cardiovascular Disease Fellowship program, an assistant professor of cardiology at the Houston Methodist Academic Institute and Weill Cornell Medical College, and an assistant clinical member of the Houston Methodist Research Institute.

Dr. Faza is a fellow of the American College of Cardiology, American Society of Echocardiography, and Society for Cardiovascular Angiography and Interventions. She has served on the American College of Cardiology’s Curriculum Design Committee and is currently a member of the College’s Competency Management Committee. She also is a member of the American Society of Echocardiography’s Public Relations Committee and serves as the Social Media Chair for the Texas Chapter of the American College of Cardiology. She is a member of the editorial boards of the *Methodist DeBakey Cardiovascular Journal* and *the Journal of the American College of Cardiology - Case Reports*.

## Stephen H. Little, MD

**Figure:**
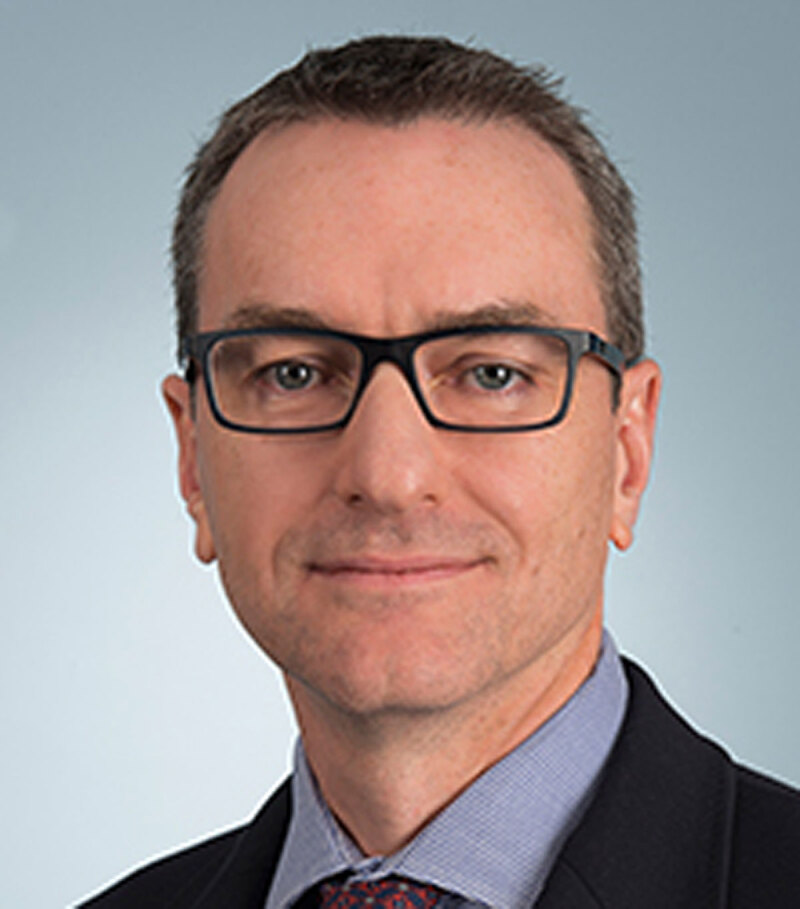


Dr. Little is a cardiologist with the Houston Methodist DeBakey Heart & Vascular Center and program director of Houston Methodist’s Cardiovascular Fellowship program. He also is a professor of medicine at Weill Cornell Medical College and adjunct professor at Rice University’s Department of Bioengineering.

He is a fellow of the Royal College of Physicians and Surgeons of Canada, the American College of Cardiology, and the American Society of Echocardiography. He is currently the president of the American Society of Echocardiography, having served on its Board of Directors and chairing the guidelines committee.

